# Supplementary material for: The interactome of intact mitochondria by cross-linking mass spectrometry provides evidence for coexisting respiratory supercomplexes
Source: Mol Cell Proteomics. 2017 Dec 8;17(2):216–32. doi: 10.1074/mcp.RA117.000470 (PMC5795388; doi:10.1074/mcp.RA117.000470)
Supplement: Supplemental Data [file supp_RA117.000470_133922_0_supp_23978_fzffwf.zip › spectra_annotation/mito_spectra_annotation/231-1-4-1-11-1.pdf]

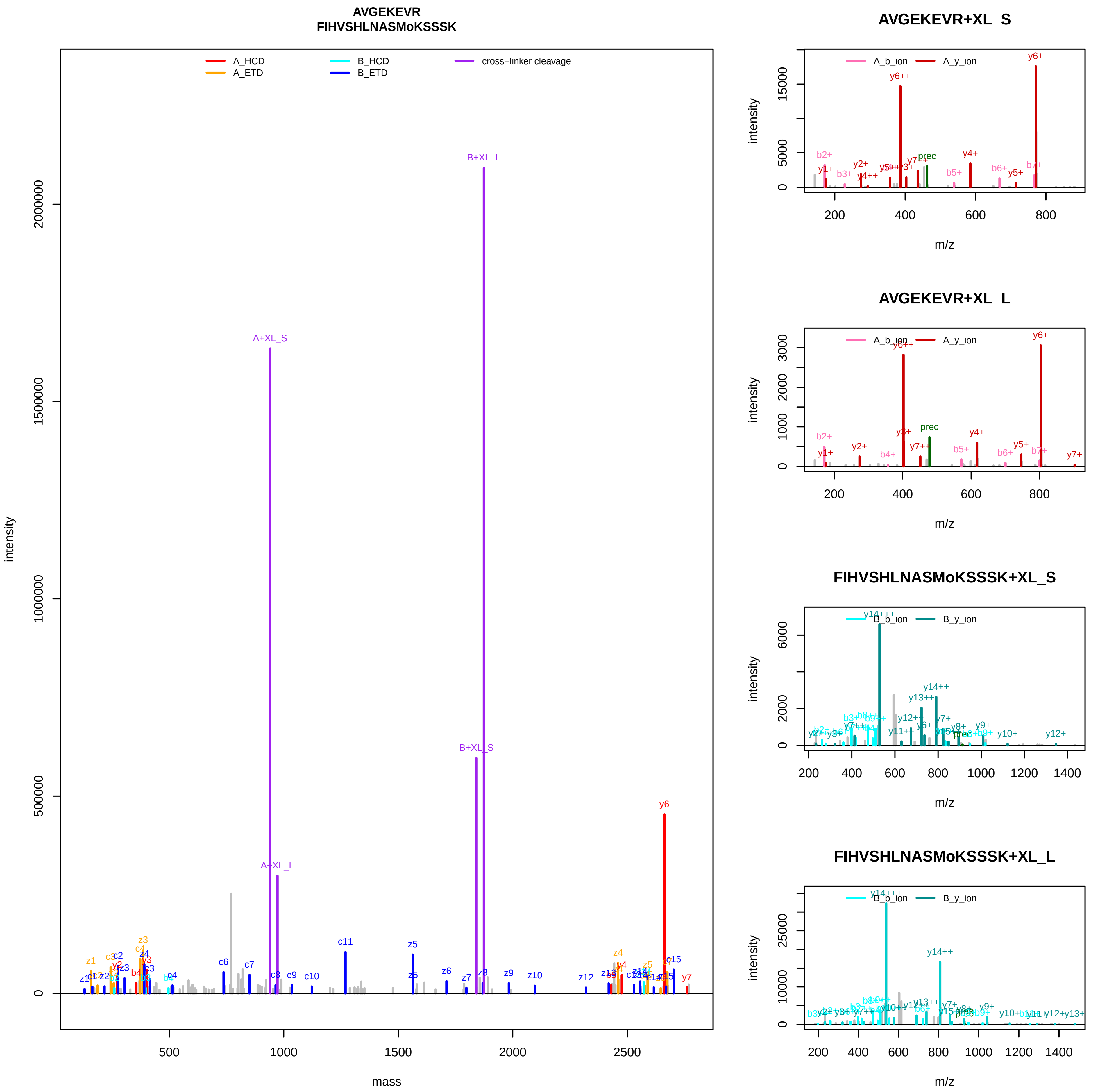
AVGEKEVR
FIHVSHLNASMoKSSSK
A_HCD
A_ETD
B_HCD
B_ETD
cross−linker cleavage
intensity
mass
B+XL_L
A+XL_S
B+XL_S
A+XL_L
y6
c11
z5
c15
y7
AVGEKEVR+XL_S
A_b_ion
A_y_ion
y6+
y6++
prec
y4+
intensity
m/z
AVGEKEVR+XL_L
A_b_ion
A_y_ion
y6+
prec
y4+
y7+
intensity
m/z
FIHVSHLNASMoKSSSK+XL_S
B_b_ion
B_y_ion
y14+++
y14++
y13++
y12+
intensity
m/z
FIHVSHLNASMoKSSSK+XL_L
B_b_ion
B_y_ion
y14+++
y14++
intensity
m/z
